# Transoral robotic supraglottic partial laryngectomy: report of the first Brazilian case^☆^

**DOI:** 10.1016/j.bjorl.2016.01.016

**Published:** 2016-05-03

**Authors:** Claudio Roberto Cernea, Leandro Luongo Matos, Dorival de Carlucci Junior, Fernando Danelon Leonhardt, Leonardo Haddad, Fernando Walder

**Affiliations:** a Universidade de São Paulo (USP), Faculdade de Medicina, Disciplina de Cirurgia de Cabeça e Pescoço, São Paulo, SP, Brazil; b Hospital Israelita Albert Einstein, São Paulo, SP, Brazil; c Universidade Federal de São Paulo (UNIFESP), Disciplina de Otorrinolaringologia - Cirurgia de Cabeça e Pescoço, São Paulo, SP, Brazil

## Introduction

In the past decade, we have witnessed the introduction and dissemination of transoral robotic surgery for the treatment of tumors, mainly of the oropharynx and larynx. The use of robotic surgery improves visualization of the operative field due to its three-dimensional image and enhances the surgeon's dexterity due to bimanual control of the robotic arms. Furthermore, the assistant contributes with suction and tissue traction, which leads to the use of four instruments during surgery, something impossible during a transoral resection through laryngoscopy, for instance.^1^ Therefore, the technique makes the approach truly minimally invasive, especially in the case of supraglottic partial laryngectomy, in which the conventional open approach inevitably leads to protective tracheostomy and feeding tube use, sometimes for prolonged periods. The robotic access, however, allows for early feeding without the need of a tube, and also eliminates the need for tracheostomy in many cases, as the rates of aspiration, fistulas, or other complications are significantly reduced when compared with conventional surgery and with oncologic and functional results that are quite similar between the two techniques.^2^

Therefore, this study reports the first case of supraglottic partial laryngectomy performed by transoral robotic surgery in Brazil, as well as documents the late oncologic and functional results (Approved by the Research Ethics Committee under No. 228/14).

## Case report

A 57-year-old female patient was evaluated for a four month complaint of odynophagia; she was a long-term smoker (30 pack-years) and a non-alcoholic. Physical examination revealed no lesions at the oroscopy and no palpable cervical lymph nodes. The nasofibrolaryngoscopy identified a large vegetating lesion affecting the entire epiglottis and extending to the left aryepiglottic fold, but not affecting the arytenoid fold or the left ventricular fold; both vocal folds were still mobile.

**Figure 1:**
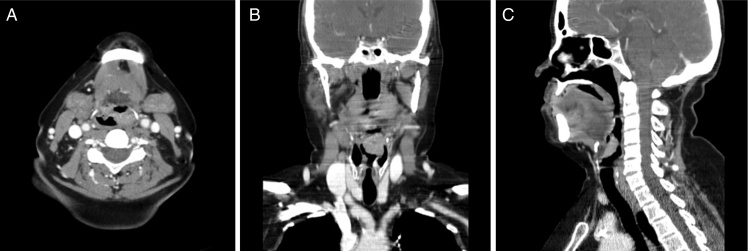
Computed tomography depicting a vegetative lesion in the epiglottis and affecting the left aryepiglottic fold in the axial (A), coronal (B), and sagittal (C) views.

An incisional biopsy revealed that the lesion was a moderately differentiated squamous cell carcinoma (SCC). Assessment by computed tomography (Fig. 1) showed that the lesion had limits compatible with the laryngoscopy, without pre-epiglottic space involvement and without cervical lymph nodes suggestive of metastases. There was no evidence of pulmonary metastases; the search for a second primary tumor through high digestive endoscopy with chromoendoscopy was negative, and the cancer was staged as T2N0M0 (stage II).

**Figure 2:**
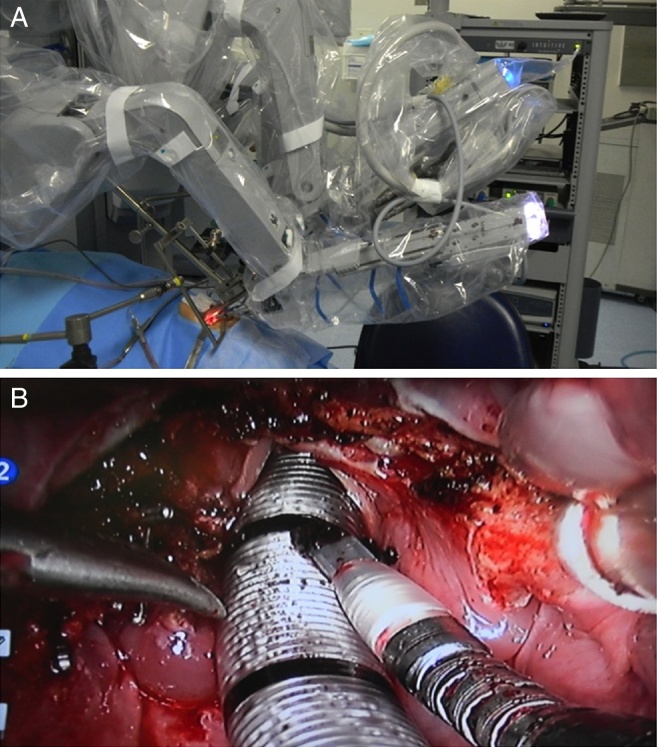
Intraoperative period. (A) Positioning of robotic arms and optical sensor; (B) surgical wound appearance after supraglottic laryngectomy.

The patient then underwent a transoral robotic supraglottic partial laryngectomy using the *daVinci SI Surgical System*^®^ (Intuitive Surgical^®^; Sunnyvale, California, United States) equipment (Fig. 2). The procedure was uneventful, lasted 158 minutes, had a 50-mL blood loss and the resection had clear intraoperative frozen section margins. There was no need for tracheostomy and the patient was extubated in the operating room under endoscopic view. Also, the use of a parenteral feeding tube was not necessary, and the patient received a thickened liquid diet on the second postoperative day, without evidence of aspiration. The length of hospital stay was three days. Definitive anatomopathological analysis disclosed a moderately differentiated SCC without perineural or angiolymphatic invasion with margins free of tumor.

After 24 postoperative days, the patient underwent uneventful selective cervical dissection of levels II, III, and IV bilaterally and histopathological analysis found no metastases in 57 dissected lymph nodes; she was discharged within 72 hours.

There was no indication for adjuvant treatment, and the patient remains on outpatient follow-up, with no evidence of disease, with a normal diet and no voice alterations at 42 months of follow-up.

## Discussion

Since the first published work by Weinstein in 2007^3^ with the description of the first three cases, other centers began to perform supraglottic laryngectomy using the transoral robotic approach, but the number of reported cases is still low. The largest series in the literature included 84 surgeries performed in seven French services.^1^ The authors demonstrated that the mean time of parenteral tube use was eight days and 24% of patients resumed oral intake 24 hours after the procedure. Only 24% of patients required a tracheostomy, but there was aspiration pneumonia in 23% of cases, including one death for that reason. Postoperative bleeding occurred in 15 patients and 51% of the patients required adjuvant radiotherapy due to the anatomopathological findings, but there is no description in this study of the oncologic outcomes in these patients.

Therefore, a systematic review in the Medline database until September 2015 (using the key words [“laryngectomy” and “robotic surgery”]) was performed, and it retrieved 11 articles,^1^, ^3^, ^4^, ^5^, ^6^, ^7^, ^8^, ^9^, ^10^, ^11^, ^12^ totaling 176 cases, in addition to the patient reported herein (Table 1). It was observed that most of the included patients had tumors at an early stage (stages I and II) and that the surgery was performed with free margins in most cases, with few complications. The need for tracheostomy and a parenteral feeding tube was variable, but brief, in most cases. The need for adjuvant therapy was low and oncologic results showed no cases of local recurrence, demonstrating the safety of the method.

**Table 1:** Results of the systematic review of published cases of robotic supraglottic partial laryngectomy due to squamous cell carcinoma.

Study	*n*	Age (years)	Primary lesion	cT	cN	Neck Dissection	Margins
Weinstein 2007^3^	3	59	Supraglottic	T2	N0	Yes	Free
		59		T2	N0	Yes	Free
		69		T3	N0	Yes	Free

Alon 2012^4^	7	72	Supraglottic	T2	N1	Yes	Free
		51		T1	N0	Yes	Free
		45		T3	N0	Yes	Free
		57		T2	N0	Yes	Free
		67		T2	N2b	Yes	Free
		67		T1	N1	Yes	Free
		71		T2		Yes	Free

Ozer 2012^10^	13	58 (mean)	EP (100%)	1 T1	11 N0	Yes (all)	Free (all)
			AEF (76.9%)	10 T2	2 N2b		
			VF (23%)	2 T3			
			BT (23%)				
			EP (15.3%)				
			PS (15.3%)				

Ansarin 2013^5^	10	68 (mean)	Supraglottic	2 T1	6 N0	40%	Positive in 40% of patients
				6 T2	4 N+		
				2 T3			

Lallemant 2013^8^	10	64	EP/AEF	T2	N2c	Yes	Free
		67	EP	T2	N1	Yes	Free
		75	EP	T1	N0	Yes	Free
		63	EP/AEF	T1	N0	Yes	Free
		60	EP/AEF/BT	T2	N2b	Yes	Free
		50	VF	T1	N0	Yes	Free
		59	AEF	T1	N0	Yes	Positive
		60	AEF/VF/AT	T2	N0	Yes	Free
		67	AT/AEF	T2	N0	Yes	Free
		51	AEF/VF	T2	N0	Yes	Positive

Mendelsohn 2013^9^	18	ND	Supraglottic	5 T3/4a		6 NDis	Free in all cases
				13 T1/2		12 SL	

Park 2013^11^	16	66 (mean)	10 EP	7 T1	9 N0	Yes (No for 2 cases of EP T1N0)	Positive in 2 cases (12%)
			4 AEF	5 T2	3 N1		
			2 VF	4 T3	3 N2b		
					3 N2c		

Durmus 2014^6^	1	45	EP/VF	T2	N0	Yes	ND

Kayhan 2014^7^	13	60 (mean)	Supraglottic	4 T1	9 N0	Yes (all)	Free in all cases
				9 T2	3 N2c		
					1 N3		

Perez-Mitchel 2014^12^	1	68	VF	T2	N0	No	Positive

Razafindranaly 2015^1^	84	59 (mean)	Supraglottic	29 T1	54 N0	67 cases (80%)	Positive in 8 cases (9.5%)
				46 T2	11 N1		
				9 T3	4 N2a		
					9 N2b		
					5 N2c		
					1 N3		


Study	Perioperative complications	TCT (days)	ENS/GTM (days)	Hospital length of stay (days)	Adjuvant treatment	Local recurrence
Weinstein 2007^3^	No	–	–	3	–	ND
	No	–	–	8	–	
	No	–	–	5	CT + RT	

Alon 2012^4^	No	–	–	ND	–	No
	No	–	56		–	No
	Burning	4	38		–	No
	No	45	45		–	No
	No	Dependent	GTM RT		RT	No
	No	–	–		–	No
	No	–	GTM RT		RT	No

Ozer 2012^10^	1 conversion to negative margins	17 (1 case)	40 (1 case)	3.9 (mean)	RT (2 cases N+)	No (median of 6.8 months)

Ansarin 2013^5^	None in 10 cases	90%	70% (mean 12 days)	13 ± 6 days (mean)	70% (5 CT + RT; 1 new surgery for free margins; 1 RT)	No (median of 5 months)

Lallemant 2013^8^	No	4	5	ND	CT+RT	No
	No	–	2 years		RT	No
	No	–	21		–	No
	No	–	–		–	No
	No	–	20		CT+RT	No
	Bleeding	–	–		–	No
	No	–	2		RT	No
	No	–	8		–	No
	No	3	5		–	No
	No	3	4		–	No

Mendelsohn 2013^9^	None in 18 cases	None	0% GTM (ENS: ND)	11 (median)	10 CT+RT	No
Park 2013^11^	None	Yes (all cases; mean 11.2 days)	Yes (all cases; mean 8.3 days)	13.5 (mean)	Yes in 8 cases (RT 3 cases, CT+RT 5 cases)	No (mean of 20.3 months)
Durmus 2014^6^	No	–	–	ND	–	ND
Kayhan 2014^7^	2 cases of aspiration pneumonia	1 case	Yes (all; mean 21.3 days)	Yes (all; mean 8 days)	5 CT + RT	(mean of 14.1 months)
Perez-Mitchel 2014^12^	No	3 (OTI)	14	5	–	No (median of 30 months)

Razafindranaly 2015^1^	1 conversion	24 cases (24%; mean 8 days; 1 case dependent on TCT)	64 cases (76%; mean of 8 days; 1 case of permanent GTM)	15.1 (mean)	CT+RT in 43 cases (51%)	ND
	16 cases of bleeding					
	19 cases of aspiration pneumonia					
	1 pharyngocutaneous fistula					

–, procedure not performed; AEF, aryepiglottic fold; AT, arytenoid; BT, base of tongue; CT, chemotherapy; ENS, Enteral nutrition support?; E.P, epiglottis; GTM, gastrostomy; NDis, neck dissection; ND, no data; OTI, orotracheal intubation; PS, pyriform sinus; RT, radiotherapy; SL, sentinel lymph node screening; TCT, tracheostomy; VF, ventricular fold; VF, vocal fold

In this case, some aspects are noteworthy and were later verified by other studies summarized here: the patient had an uneventful postoperative period, in addition to very satisfactory oncologic and functional results. The desire to provide the patient's late follow-up status led to the delay in reporting the present case.

## Conclusion

This case describes the viability of supraglottic partial laryngectomy by transoral robotic approach, with good postoperative evolution and early rehabilitation. It is therefore a safe method, with very satisfactory oncologic and functional results.

## Conflicts of interest

The authors declare no conflicts of interest.
